# A Phased Approach for Assessing Combined Effects from Multiple Stressors

**DOI:** 10.1289/ehp.9331

**Published:** 2007-01-24

**Authors:** Charles A. Menzie, Margaret M. MacDonell, Moiz Mumtaz

**Affiliations:** 1 Exponent, Inc., Winchester, Massachusetts, USA; 2 Argonne National Laboratory, Argonne, Illinois, USA; 3 Agency for Toxic Substances and Disease Registry, Atlanta, Georgia, USA

**Keywords:** combined effects, cumulative risk assessment, GIS, multiple stressors, phased approach, stressor identification

## Abstract

We present a phased approach for evaluating the effects of physical, biological, chemical, and psychosocial stressors that may act in combination. Although a phased concept is common to many risk-based approaches, it has not been explicitly outlined for the assessment of combined effects of multiple stressors. The approach begins with the development of appropriate conceptual models and assessment end points. The approach then proceeds through a screening stage wherein stressors are evaluated with respect to their potential importance as contributors to risk. Stressors are considered individually or as a combination of independent factors with respect to one or more common assessment end points. As necessary, the approach then proceeds to consider interactions among stressors. We make a distinction between applications that begin with effects of concern (effects based) or with specific stressors (stressor based). We describe a number of tools for use within the phased approach. The methods profiled are ones that have been applied to yield results that can be communicated to a wide audience. The latter characteristic is considered especially important because multiple stressor problems usually involve exposures to communities or to ecologic regions with many stakeholders.

In this article we present a phased approach for evaluating the effects of physical, biological, chemical, and psychosocial stressors that may act in combination. We outline basic steps in the process within which various analytical and modeling tools can be used. Although risk assessment applications can involve a range of phases or steps (two to five are common in regulatory programs), the key idea proposed here is the iterative development of information appropriate to answering the problem at hand. Rather than beginning with a concerted effort to conduct full-scale risk assessments of multiple stressors across all possible combinations, we suggest that the phased inclusion of multiple stressors and associated interactions be guided by the needs of the assessment.

Organizing the problem in phases is a practical approach. Useful insights can best be gained by making problems as tractable as possible so that a shared understanding can be achieved by the public, risk managers, and risk analysts. When dealing with multiple stressors and effects, the effort to reduce the risk problem is complicated by the fact that the role of some stressors may not be obvious at the outset; thus, some could be dropped during the simplification process. Thus, applying a phased approach with modular or nested models and rolling checkpoints to revisit screened components helps assure appropriate consideration of stressors and effects. The practical aspects we present in this article are complemented by technical papers on biological markers to measure cumulative effects (Ryan et al. 2007), effects of differential exposure (Sexton and Hattis 2007), and effects of differential preparedness and resilience (deFur et al. 2007).

We envision two circumstances where information on the combined effects of multiple stressors can inform environmental management decisions. The first is when the causes of observed effects on human health or the environment are unknown or poorly understood and the possibility of combinations of stressors is suspected. This is essentially an epidemiologic approach directed at unraveling causes of observed conditions. These retrospective approaches are referred to as effects-based assessments (Ferenc and Foran 1999) or population-based, as described in the U.S. Environmental Protection Agency “Framework for Cumulative Risk Assessment” (U.S. EPA 2003a). The second circumstance is when a stressor is known or suspected to combine with or be influenced by other stressors or conditions. Examples of the latter include emerging technologies and products that are not well understood, such as nanomaterials. These are typically prospective analyses and are referred to as stressor-based assessments.

Three criteria were used to profile tools for use within the phased approach: the tool is readily available, it has been applied to the problem or similar problems, and it yields results that can be communicated to a wide audience. The latter characteristic is considered especially important because multiple stressor problems usually involve exposures to communities or to ecologic regions with many stakeholders.

Health and environmental risk assessments have targeted chemical hazards and certain physical stressors for decades; therefore the tools for these applications are well developed. For this reason these applications are the main focus of this article. More recent assessments extend to broader interactions, such as impacts of climate change and other factors such as infrastructure, on endemic and pandemic diseases. For example, climate change assessments that guide public health planning consider direct and indirect effects on pathogen viability (such as encephalitis or West Nile virus), altered temporal and spatial scales for vectors (such as mosquitoes) and home ranges of reservoirs (such as horses or birds). These assessments apply the basic concepts and many of the same tools highlighted in the discussions that follow, and they provide further valuable insights for understanding the wide range of stressors and effects involved in local, regional, and global risk issues.

Because evaluating the combined effects of multiple stressors can be complex, early phases of the assessment should strive to achieve clarity and focus that can be carried throughout the evaluation. This is accomplished, in part, through the development of conceptual models and selection of integrated assessment end points.

## The Conceptual Model

**Figure 1:**
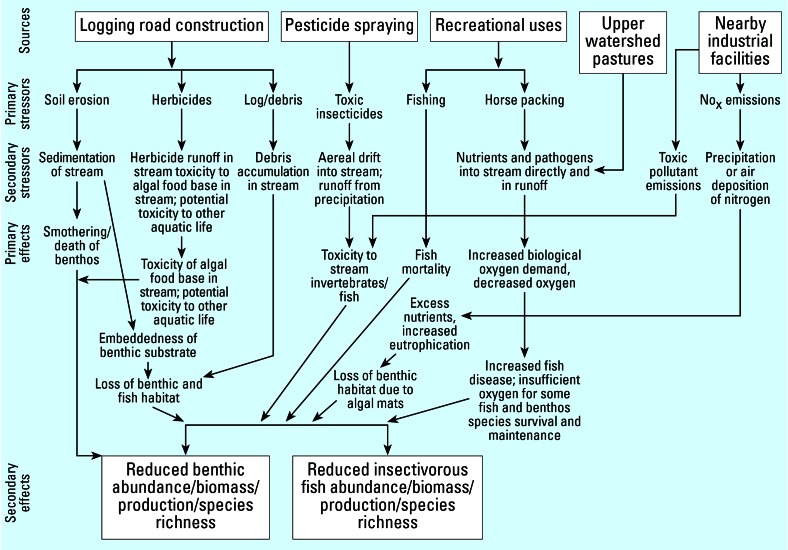
Case-specific conceptual model for aquatic biota assessment. NO_x_ (nitrogen oxide). This figure illustrates how stressors may combine to cause effects upon aquatic biotal and includes short explanations on how the stressors cause the effects. Figure reproduced from U.S. EPA (2004a).

Developing a conceptual model is critical to any assessment of combined effects from multiple stressors. As shown in Figure 1, conceptual models typically provide a visual representation of the stressors along with their direct and indirect effects here for an aquatic ecologic assessment. As the figure indicates, such models can include short explanations regarding the nature of effects and possible interactions. This example captures both physical and chemical stressors and also incorporates a social component, as reflected in the recreational use category.

**Figure 2:**
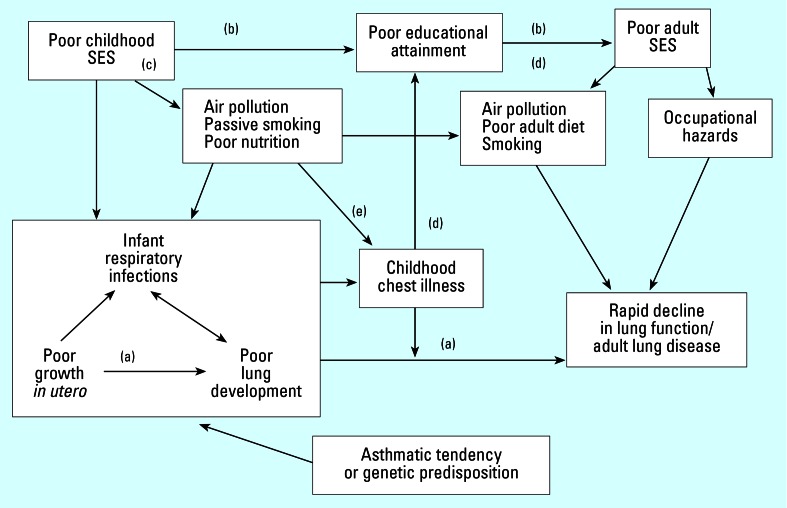
Conceptual model for assessing chronic respiratory effects. SES, socioeconomic status. Included are biological and psychosocial exposures acting across the life course that can influence the health outcome; letters indicate predominant pathway type: (*a*) biological, (*b*) social, (*c*) sociobiological, and (*d*) biosocial. Reproduced from Ben-Shlomo and Kuh (2002) with permission from Oxford University Press.

Similarly, conceptual models for human health assessments can be structured to reflect the combination of stressors and processes by which diseases may emerge, as shown in Figure 2, which depicts a life-course approach to chronic disease epidemiology (Ben-Shlomo and Kuh 2002). The figure illustrates a conceptualization for adult respiratory disease and impaired lung function, depicting how early life exposures and other factors can lead to later disease. Incorporating the temporal component, the model captures biological and chemical as well as socioeconomic factors that can contribute to that health outcome.

Because assessments of multiple stressors tend to be complex, a key challenge is balancing detail and clarity within the conceptual model(s). One approach for accomplishing this balance is to use multiple models (Gentile et al. 2001). A second is to use modules that can be combined (Suter 1999), and a third is to use interactive hierarchical (nested) computer-based models that can be expanded and contracted to show overviews of the problem as well as the richness of detail associated with specific aspects of each level of the analysis.

Conceptual models of systems with multiple stressors can also be developed as Bayesian networks that subsequently can be applied to analyze problems. Variables such as stressors in Bayesian networks are commonly treated as discrete, with influences represented by conditional probability tables. The networks can be used to help describe relationships among the stressors and identify which stressors are important and which effects may result from the combinations of stressors. The graphical structure of a Bayesian network integrates cause and effect relationships into an articulated series of conditional relationships, each of which can be independently quantified using a submodel suitable for the type and scale of information (Borsuk et al. 2004).

## Defining Common Receptors and End Points

Combined effects are considered in terms of common receptors and end points. These serve as common denominators for aggregating and evaluating the combination of effects from multiple stressors as well as for considering how stressors and effects may interact with one another. We use the term “assessment end point” to refer to the combination of the receptor and effect end point (after U.S. EPA 1992). Human and ecologic receptors are most commonly defined as individuals, communities, or populations. In the case of human health risk assessments, an evaluation of multiple stressors might typically involve a particular worker population or a particular community. For ecologic or environmental assessments, receptors might also include habitats, particular ecologic systems, or larger regions. Receptors can also include ecologic processes such as the sequestration of carbon by oceans or forests or the cycling of nutrients.

An end point, as used here, refers to the condition being evaluated. This could be expressed as an incidence, rate, or status of an attribute of the receptor. Examples include mortality rates and other loss or process rates, the incidence of a disease such as cancer or asthma, reproductive or developmental effects, abundance (for populations), and acreage (for habitats). An evaluation of combined effects could involve one or more assessment end points.

The assessment end points need to be chosen and expressed in a manner that is sufficiently specific for the issues being evaluated and amenable to aggregating the combined effects of multiple stressors. An example of an assessment end point that is overly broad is health effects among the population in the City of Chicago. Although this has some specificity with regard to the location, there are many different types of health effects and considerable variability among subpopulations. Consequently, the analysis can be improved by generating assessment end points that are more specific in terms of the type of health effect and the particular subpopulation and/or age group. An example would be incidence of asthma in children living in a particular part of Chicago. As assessment end points are made more specific, the need for these will become more evident. In our view, it is better to have several specific assessment end points (and associated evaluations of combined effects) rather than one very general and comprehensive assessment end point.

The types of assessment end points identified above should be familiar to human health and ecologic risk assessors. However, there may be cases for which it is useful to translate the various elements of the human and ecologic systems into a common metric or environmental currency (U.S. EPA 2003b, 2004a). For example, the combined effects of multiple stressors on human health could be expressed in terms of the disability-adjusted life year (DALY) or the quality-adjusted life year (QALY), as summary measures of population health (Prüss-Üstün et al. 2003). The DALY combines the number of years of healthy life lost because of premature mortality and disability. The QALY takes into account both quantity and the quality of life. It is the arithmetic product of life expectancy and a measure of the quality of the remaining life years. DALY and QALY measures are used in health economics to inform policymakers. As such, they can provide a useful basis for aggregating the combined effects of multiple stressors at the level of a population.

Another common currency approach involves the use of emergy units. These translate different forms of energy within the system (including physical structures and processes) into a common physical currency (Cambell 2001). The combined effects of multiple stressors can be evaluated in terms of how they influence the overall emergy state of the system. Emergy units have been used to evaluate watersheds (Tilley and Swank 2003), resources in West Virginia (Campbell et al. 2005) and Brazil (Safonov et al. 1998), and in the biosphere (Brown and Ulgiati 1999).

## Community and Stakeholder Involvement

People are affected by multiple stressors in the environment, both directly or indirectly. Because there are often inequities in conditions and exposures across communities, the issue of health risks from multiple stressors has been the focus of much attention from the perspective of environmental justice (National Environmental Justice Advisory Council 2004; U.S. EPA 2000b). Community involvement is a critical aspect of developing a shared understanding of the problem, for without this understanding little progress can be made toward sustainable solutions. Community involvement is also critical to regional and watershed assessments that deal with broader environmental quality issues. The examples presented in this article have typically involved formal community and stakeholder involvement processes, and steps to include representatives are critical to the process. The evaluation of combined effects of multiple stressors is more than a technical challenge. It must flow from a common understanding of the issues and concerns of the affected public.

## A Phased Approach for Evaluating Combined Effects

Phased approaches, also referred to as “tiered” or “iterative” evaluations, are commonly used in risk assessments as a means to balance resources against the desire to reduce uncertainty in the assessments. Evaluating the combined effects of multiple stressors can be daunting, especially as additional stressors are included in the evaluation, with an interest in examining a wide range of effects and interactions. We view the problem from the perspective of the value of information added for decision making. Therefore, we suggest an approach that begins as simply as possible but is as comprehensive as appropriate for the problem. In-depth, more detailed analysis is added only as necessary to characterize risks at a level appropriate for the management decision.

A critical aspect of a phased approach is recognizing the core elements that should be considered in the evaluation from the onset. This suggests an inclusive conceptual approach along with an initial effort to prioritize the relative importance of the various stressors. The conceptual model helps track various exposure pathways and interrelationships and can be used to indicate the relative importance of stressor and pathway combinations. In this way, it is possible to simultaneously capture the breadth of the problem as well as focusing on its key aspects.

This phased approach has the following elements:

- Develop a conceptual model sufficient to bound the problem; include all relevant stressors and describe how they might act in combination (see Figure 1 for an example).
- Screen stressors to arrive at an appropriate and manageable number for the problem; this is a focusing exercise. Other stressors and pathways can be represented in the conceptual model, but resources are directed to understanding the stressor and pathway combinations considered to have the greatest potential effects. Retain screened stressors on a watch list for subsequent checks after more information is developed.
- Evaluate the individual effects of individual stressors to determine if any are predominantly contributing to or could contribute to the effect(s) of interest.
- Evaluate the collective effects of stressors without yet considering the potential for interactions (e.g., synergism or antagonism), and identify the potential for stressor or effect overlap, for example, based on common properties or temporal and spatial and temporal links.
- Evaluate the combined effects of stressors, taking into account potential interactions and considering qualitative to quantitative methods, depending on the information available.

Key to the iterative process is revisiting these steps at intermediate stages throughout the assessment to assure that contributing stressors, influencing factors, and effect end points are integrated so that combined effects and primary risk contributors can be well characterized to the level existing knowledge allows. The following discussion describes how this approach can be applied to effects-based and stressor-based assessments.

### Effects-based assessments

Effects-based assessments start with the effect(s) of concern. Those effects might include elevated cancer levels or other health conditions in a community or workforce, or observed changes in the biota of a stream or forest. One or more stressors may be involved, and the types of information valuable for management decisions include the following:

- Identification of the stressors that are contributing to the observed effects.
- Information on how the stressors are causing the effect either individually or in combination (i.e., some mechanistic understanding).
- Insight into how to reduce or prevent the stressor from causing the effect or how to ameliorate the effect through other measures.
- Information on the short- and long-term implications of alternative management actions.

Table 1 provides suggested steps for proceeding with an evaluation of combined effects for an effects-based assessment. The approach begins with a conceptual model that reflects existing knowledge about the nature of the effect(s) and possible causal factors (see Figures 1 and 2 for examples). In an effects-based assessment, the receptors and the effects of interest are known, and these define the assessment end points and structure the conceptual model.

The methods and tools for an effects-based assessment are diagnostic and directed at uncovering the stressors and stressor combinations that are contributing to the observed effects.

**Figure 3:**
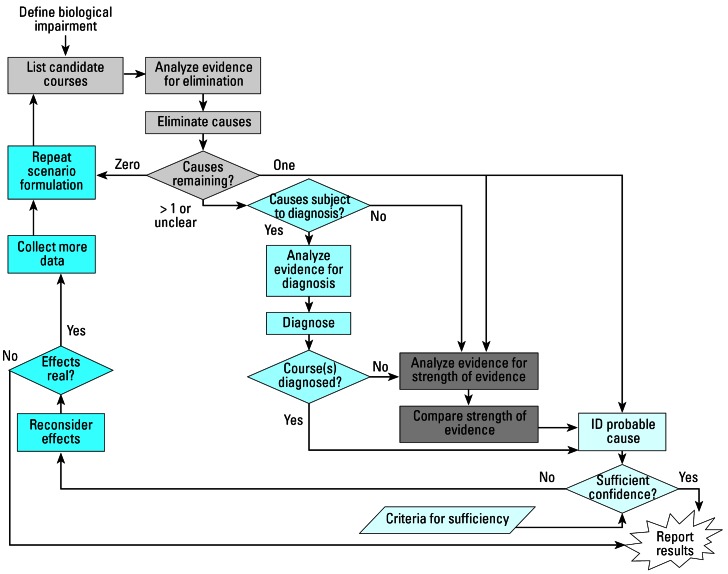
SI process. Figure reproduced from U.S. EPA (2006; http://www.epa.gov/caddis).

The U.S. EPA stressor identification (SI) process (U.S. EPA 2000a) provides a clear example of how to proceed through this process, and we believe it can be applied with adaptations to most multiple stressor problems that begin with observed effects on human health and the environment (Figure 3). The conceptual model is at the heart of the SI process and provides the means for describing the connections between candidate causes and the effects. These connections could be direct or indirect and could involve various modes of action. Work is currently underway to strengthen the conceptual modeling tools used in the process (Norton SB, personal communication). Guidance on the application of the approach is also being kept reasonably current via a website (http://www.epa.gov/caddis; U.S. EPA 2006). Importantly, the SI process is derived from a careful consideration of epidemiologic criteria put forward by Hill (1965). Further, it is easily supported by other tools designed to examine associations among variables. While the SI method currently is being used primarily to identify and differentiate causes of biological impairments in watersheds, the methodology is applicable to any ecologic or human health effects-based problem.

Effects-based approaches have long been applied to assess public health issues, with the Framingham Heart Study being a compelling example (National Heart, Lung, and Blood Institute 2002). Unraveling the causes for that effect from extensive epidemiologic studies pointed to multiple stressors, including diet and exercise approximately 40 years ago, whereas multigenerational studies have also identified the key role of genetics in recent years. A classic example for occupational epidemiology is provided by the Libby, Montana, studies that linked elevated incidences of effects (lung cancer and asbestosis) to asbestos (Agency for Toxic Substances and Disease Registry 2000; Amandus and Wheeler 1987). More recently, the value of the effects-based approach for occupational programs was illustrated by the identification of “popcorn lung” disease from worker exposures to multiple flavinoids in microwave popcorn production.

The basic concepts underlying the SI process were also applied in a short-term assessment of birth outcomes after the World Trade Center (WTC) collapse. Specific effects represented by this integrated end point have been linked to maternal exposures to polycyclic aromatic hydrocarbons (PAHs), a large number of which were released by the fires and other chemicals, as well as to stress. Results indicated babies of women who worked and lived within 2 miles of the WTC 4 weeks after the collapse weighed slightly less and were 1/3 inch shorter at birth, on average. Yet when controlled for gestation duration, newborns of women who worked within 2 miles but lived elsewhere averaged almost 2/5 inch longer (Lederman et al. 2004). Of note is that women who were in their first trimester September 11 delivered several days earlier on average regardless of location effects, which suggested a stress response. Such studies demonstrate the importance of considering multiple candidates and offer insights for chemical and psychosocial stressor combinations.

In assessing chronic disease, the life-course approach (Ben-Shlomo and Kuh 2002) appears promising as a way of evaluating the combined effects of multiple stressors with underlying biological factors. This approach assesses long-term effects on chronic disease risk related to physical and social exposures during gestation, childhood, adolescence, young adulthood, and later adult life. Included are studies of the biological, behavioral, and psychosocial pathways that operate across an individual’s life course, as well as across generations, to influence the development of chronic diseases. The approach begins with a conceptual model (Figure 2) and requires an understanding of the natural history and physiologic trajectory of normal biological systems.

Conceptually, the life-course approach is relatively straightforward. The challenge is in implementation. Nevertheless, the approach offers a framework for organizing information in a manner that enables evaluation of stressors that act throughout a lifetime and may be particularly important during critical and sensitive life stages. Case-specific and basic knowledge is often limited, and the lack of knowledge on the nature of exposures and interactions among stressors is among the most challenging aspects of elucidating stressors, pathways of exposure and causal mechanisms.

Screening-level assessments for individual or multiple stressors might also include the use of health-based or ecologically based reference systems to judge the nature and degree of departures of the system from the reference set. Examples of reference systems include national or regional databases with statistics on normative health conditions and indices of biological integrity for aquatic ecosystems. Munkittrick and McMaster (2000) have developed and applied a reference condition approach that involves measuring the accumulated environmental state of the system to evaluate multiple stressors on fish populations. The authors rely on the characteristics of the departures from the reference state to identify the candidate stressors.

**Figure 4:**
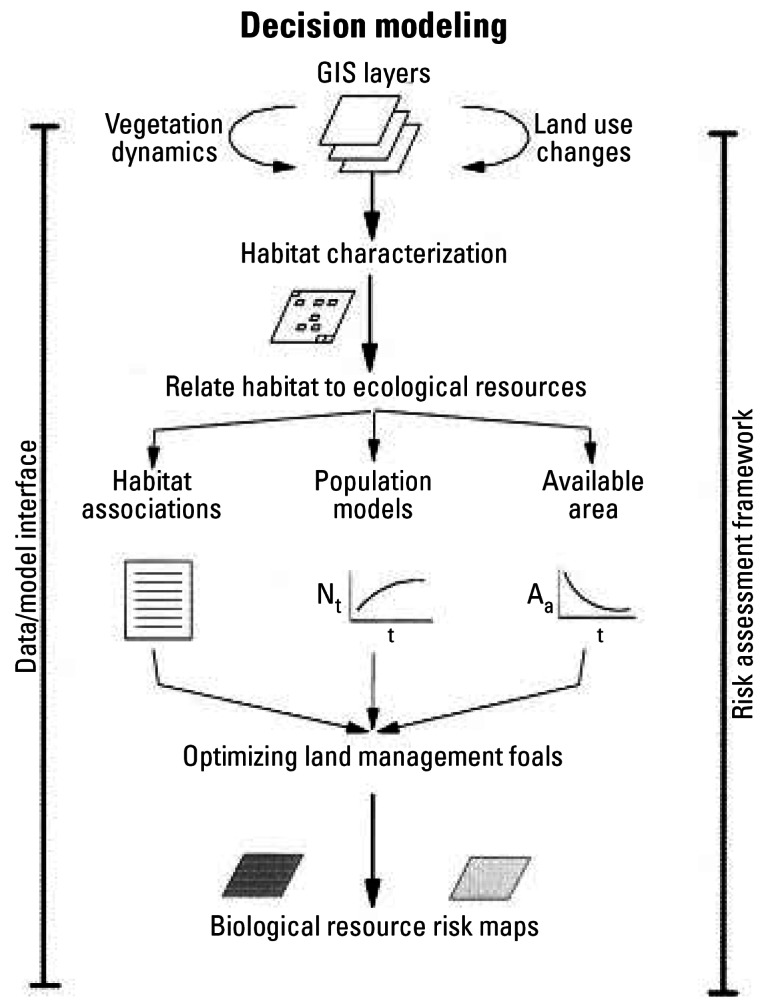
GIS-based habitat assessment (Dale et al. 1998). Figure reproduced from Dale et al. (1998) with permission from Springer Science and Business Media.

**Figure 5:**
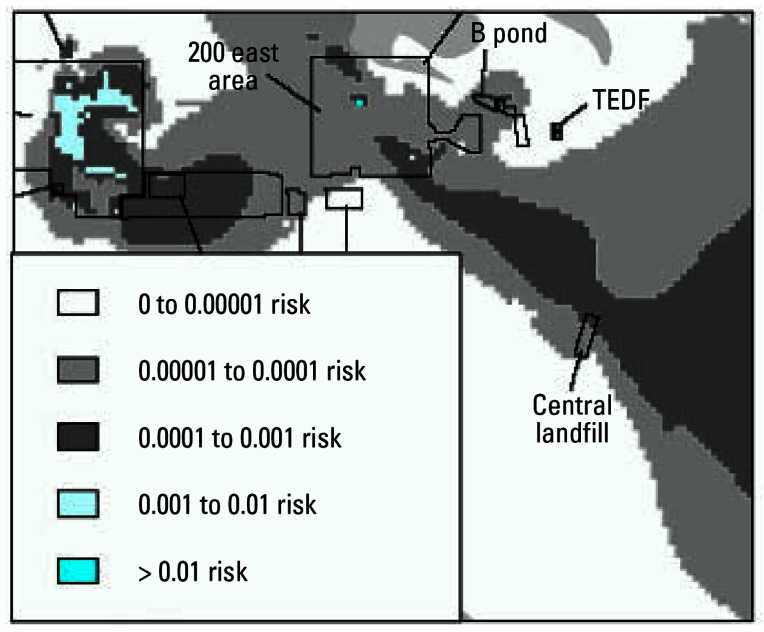
Estimated health risk contours from GIS-integrated groundwater data. Figure reproduced from Pacific Northwest National Laboratory (2002).

Where exposure groups and the candidate stressors are defined in terms of spatial boundaries, geographic methods—primarily using geographic information systems (GIS)—are among the best means for organizing the available information and relating the locations of effects on human and ecologic receptors to the locations and magnitudes of the various stressors. The potential for combined effects of multiple stressors can be evaluated by examining the overlap of stressors (extent and magnitude) where these are illustrated as layers within a GIS or other spatial framework (Landis and Weigers 2005; Zandbergen 1998). As examples, Figure 4 depicts the GIS-based concept for assessing land use impacts on habitat (Dale et al. 1998), and Figure 5 illustrates combined risk contours developed from GIS-linked ground-water data for a large radioactively and chemically contaminated site (Pacific Northwest National Laboratory 2002).

GIS can support the development and application of community-based environmental load profiles (ELPs) as developed in U.S. EPA Region 2. The ELPs are derived from indices or measures of potential exposure and provide insight into the spatial distribution of combined exposure loads over various communities (U.S. EPA 2000b). U.S. EPA Region 6 has also developed a GIS-based approach for aggregating risk factors across landscapes.

GIS tools have also played a key role in community-based cumulative health risk assessments. Monitored and modeled concentrations of air pollutants have been combined with reference toxicity values to develop toxicity-weighted hazard and risk plots, which are combined with spatial maps of demographics and disease rates to focus more detailed assessments on stressor source–effect regions identified as highest priorities based on health concerns (U.S. EPA 2004b).

Multivariate statistical methods are the most common and useful tools for exploring associations between responses and combinations of candidate stressors (Table 2) and can be used to help design studies. Results can then be evaluated to determine whether there is a predominant stressor that explains most of the variance or whether a combination of stressors needs to be considered (Ross and Davis 1990; Serveiss 2002). Statistical tools can also identify possible interactions among stressors. The utility of the various methods presented in Table 2 depends primarily on the types of data they can accommodate, their ability to either isolate stressors that are important [e.g., analysis of covariance (ANCOVA)] and take into account interactions among stressors [e.g., factorial multiple analysis of variance (ANOVA)].

**Figure 6:**
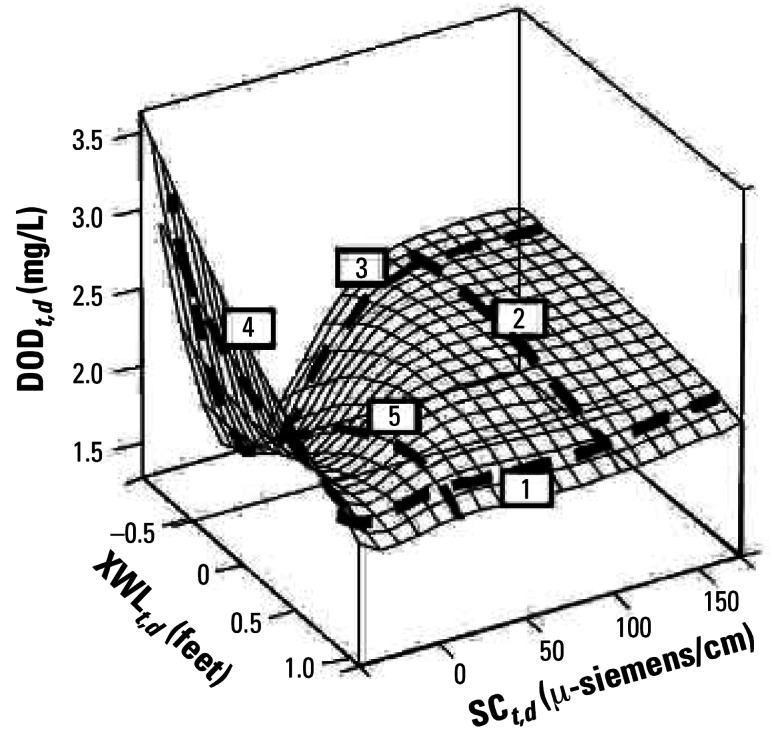
Example of a response surface for an estuary with various stressors (Conrads et al. 2002). Numbers indicate five behavioral modes mapped onto the response surface. Figure reproduced from Conrads et al. (2002) with permission from the U.S. Geological Survey.

Visual depictions of the combined effects of multiple stressors can serve as powerful communication and analytical tools. In particular, response surface modeling can be used to explore the nature of interactions among two or more stressors (Figure 6). These have been used for evaluating combinations of factors affecting human health as well as for exploring the effects of stressors on ecosystems. The ability “to see” how two or more stressors influence responses translates the underlying multivariate models into a form that can be understood by people with varying mathematical and statistical aptitudes.

Unlike statistical models, process and mechanistic models incorporate mathematical representations of underlying processes. They can be applied to effects-based assessments to help explain observed conditions and they can be used in stressor-based assessments to make predictions. Process and mechanistic models could involve any level of organization from effects at the population level (Barnthouse et al. 2000) to the combined effects of chemicals in the body (Andersen 1991; Andersen et al. 1987; Dennison et al. 2003, 2004; Krishnan et al. 2002; Yang et al. 1995). Because mechanistic elements are included, these models can be used to examine the combined effects of multiple stressors that act on the same targets or affect the same end points. Process and mechanistic models require a fundamental understanding of the nature of causes and causal interactions. As such, the development and application of these models provide a valuable framework for investigating the combined effects of multiple stressors.

To illustrate the role of fundamental mechanistic information, several causes have been suggested for cyclic fluctuations in vertebrate populations, and studies at varying spatial scales have led to different findings. Although data at a local scale indicate several trophic levels of interactions for rabbits, a large-scale regional study in northern England indicated that the population cycles of the red grouse are affected by a single trophic interaction with a parasitic nematode (Hudson et al. 1998). These results demonstrate the importance of incorporating underlying biological processes as well as spatial and temporal scales relevant to the study population and of targeting the primary contributors to observed effects when multiple factors can be at play, so results can guide practical management options.

### Stressor-based assessments

Stressor-based assessments begin with the stressors and may be initiated for several reasons, including development of criteria or guidance for a stressor; prediction of risks associated with a new policy, project, or product; and evaluation of a system in which multiple stressors of concern are present. The design of the assessment will vary to accommodate different purposes. However, the types of information that would be valuable for management decisions include the following:

- Information on the risks associated with the stressor(s) of interest individually and in combination.
- Comparative analysis of alternatives for reducing or offsetting risks.
- Information on the short- and long-term implications of alternative management actions.

Table 3 presents suggested steps for an iterative approach that begins with stressors. As with effects-based assessments, stressor-based assessments begin with a conceptual model. This model is constructed with an emphasis on how the stressors could affect receptors or exposure groups of interest either individually or in combination. For more complex problems, especially those involving a new stressor, Bayesian networks may prove particularly helpful for organizing the conceptual model and the knowledge concerning relationships among stressors, receptors, and effects.

The stressor-based approach has been applied for decades to assess risks of environmental contamination. With additivity as the default assumption for health risk assessments, doses are added via toxic equivalence or relative potency methods for chemicals considered toxicologically similar. For those expected to act independently, the method applied is response addition. Beyond a typical screening summation, end points can be segregated by target organ or system to better characterize the potential for noncarcinogenic effects. Furthermore, an interaction hazard index can be calculated when sufficient data exist for the given mixture (U.S. EPA 2001), although that situation is rare. For cancer risk estimates, more descriptive information is now included in health assessments, thus providing further context for decisions (e.g., differences in severity, treatability, and survivability, such as between thyroid and pancreatic cancer, can be important to management options).

The organization and sharing of existing knowledge are essential for supporting assessments of multiple-stressor systems, and experience with case studies is being used to develop lists of candidate stressors and effect combinations. This approach is being pursued by some states dealing with complex water quality problems. The elucidation of mechanisms of toxic (or other) action and case studies can be used to build databases that highlight the potential for interactions among stressors. For example, resources developed to support the evaluation of joint toxicity for multiple contaminant stressors include the U.S. EPA MixTox database (available from the U.S. EPA, Cincinnati, Ohio) and interaction profiles developed by the Agency for Toxic Substances and Disease Registry.

Screening of candidate stressors highlights those that should become the focus of more in-depth analysis. Other stressors and pathways can be carried on a watch list for the analysis to assure they are tracked at some level, so they can be incorporated if new information becomes available. But it is usually not necessary or appropriate to devote the same level of analysis to each stressor–pathway combination. Screening of the candidate stressors can be accomplished by applying statistical tools (Norton et al. 2002) and strength of evidence approaches such as outlined in the SI process.

In other cases, candidate stressors can be screened if their magnitudes fall below *de minimus* levels such as human health or ecologic benchmarks, which are commonly anchored to concentrations or doses but can also correspond to overall target risks. Note that benchmarks should be used carefully as they are often derived on a stressor-specific basis. Therefore, the stressor could fall below its individual threshold but still combine with other stressors to contribute to an effect. Examples include mixtures of chemicals such as certain dioxin-like compounds, divalent metals, and PAHs that are known to act on common end points and by similar toxic mechanisms. Because of the possibility of such joint effects, screening benchmarks are sometimes set below the thresholds at which potential individual effects might occur.

Matrix and ranking methods appear to be particularly useful for organizing stressor-based assessments of combined effects. These methods can make use of disparate qualitative and/or quantitative information that often typifies what is available for the various stressors (Bryce et al. 1999; Ferenc and Foran 1999; Landis and Wiegers 1997; Zandbergen 1998). Matrix methods offer a systematic way for organizing available information. Professional judgment is used to guide the analyses, commonly involving small groups of experts and discussions with stakeholders. The goal is to identify stressors or combinations of stressors that are most likely to affect or are affecting environmental conditions. Matrix methods can also be used to examine potential interactions among stressors. Matrix and ranking approaches can be applied readily to various scales of biological organization from populations to ecosystems.

**Figure 7:**
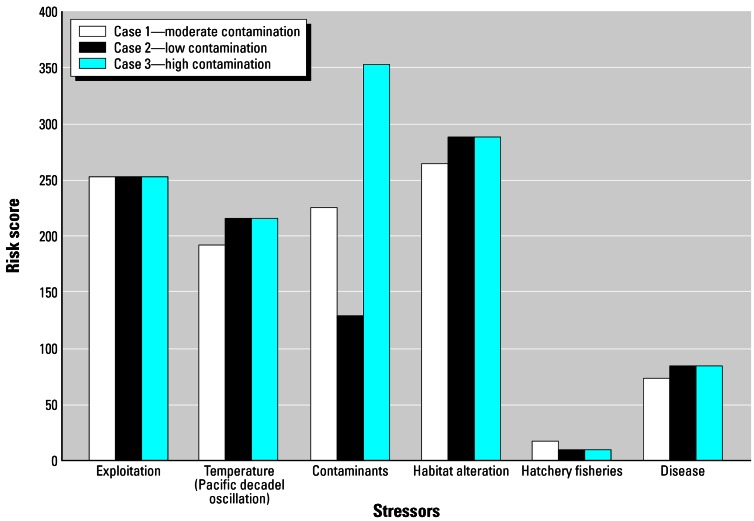
Example output from the relative risk model (RRM) illustrating combined stressor loadings (Landis et al. 2004).

The relative risk model (RRM) is an example of a matrix and ranking method that has been broadly applied (Landis 2005; Landis et al. 2004; Luxon and Landis 2005; Moraes et al. 2002). The method offers promise as a way to structure analysis of combined effects for stressor-based evaluations. RRM relies on ordinal ranks for classifying the relative importance or magnitude of sources of stressors, effects, and the estimate of impacts. The use of ranks makes it possible to combine measures that are in very different units. For example, a chemical and a physical stressor (e.g., temperature) can be combined with respect to how they might alter habitat. The results can be presented graphically to portray the accumulated stressor load with respect to an assessment end point (Figure 7).

GIS-based approaches have been used to forecast the combined effects of multiple stressors (U.S. EPA 2004b; Zandbergen, 1998). One such approach—Alternative Futures Analyses—relies on GIS-based tools to examine how landscape changes translate into changes in the conditions of watersheds (Kepner et al. 2004; U.S. EPA 2002). The landscape changes are converted to changes in physical and chemical stressors that can act together to alter conditions. This analysis is accomplished by underlying process models and through the use of professional judgment. The stressors are then related to spatially-explicit outcomes in overall condition. Because the results are presented as maps that show the net changes in conditions, they are accessible by a wide audience. Other examples of spatially explicit approaches that include multiple stressors are *a*) the Land Use Evolution and Assessment model (LEAM), which has recently been adapted to evaluate land use and environmental and economic impacts at military installations, and *b*) cumulative habitat and watershed impact approaches (Dale et al. 1998; Wickwire et al. 2004).

Statistical models of multiple stressors can be derived to support predictive tools that can be applied for other systems. Multiple and logistic regressions can be used to identify the relative contributions of multiple stressors to observed effects, and the resulting equations can be used to predict combined effects. Diamond and Serveiss (2001) and Potter et al. (2004) have used regression equations to examine the explanatory power of variables in watersheds. Norton et al. (2002) used principal components analysis to evaluate the relative importance of 18 stressors, then used the first six stressor factors (various combinations of the original 18) within a multiple regression model.

These regression models are statistical rather than mechanistic. So although they can work well for the system from which they are derived and do provide insight into the potential importance of various stressors under those conditions, their reliability diminishes when they are applied to conditions that fall outside the bounds of that original system. While they may still provide insight in such cases, the uncertainty associated with the resulting predictions increases with the degree of departure from the original conditions.

Process and mechanistic models simulate exposure–response relationships for stressors by representing the underlying processes and/or physicochemical characteristics, which are translated into equations. Because these models are built on knowledge of causal relationships, they can be adapted to new systems and problems. These models have been used to evaluate processes within organisms, populations, and ecosystems. Because of their potential predictive power, these models offer a means of representing the combined effects of multiple stressors, as illustrated in the discussion of effects-based assessments.

## Next Steps

Much is unknown about the combined effects of multiple stressors on human health and ecologic conditions over time. Much more research is needed to produce the data and methodologies for more definitive assessments that integrate across types of stressors and effects. This will be an evolving process, driven by emerging needs for health and environmental management policies and decisions. Gaps identified during preparation of this article suggest that further information in the following areas could help improve our understanding of cumulative risks to guide next steps. These are organized by general analytical phase, from scoping and screening to risk characterization and decision making. Basic research is a priority, as it provides the foundation upon which tools and criteria are based. In keeping with the iterative theme, the latter are also a priority to guide basic research toward information that addresses practical decision needs:

- Screening and grouping approaches to determine when detailed cumulative assessments are useful for decisions and to focus their scope by addressing priority stressors and effects, considering different types of sources, settings, and stressor and receptor properties. For example, for multiple contaminants this would include screening and grouping approaches to jointly evaluate partitioning and transformation over time, bioavailability and mode/mechanism of action, interactions, and differential responses.
- Methods to define criteria and thresholds when detailed cumulative assessments are warranted (such as benchmarks or hazard indices based on groupings that consider more than a primary target organ/system). This would also consider methods to define appropriate index stressors across a range of scenarios and to quantify relative potencies for multiple stressors with different modes of action.
- Integrated approaches to test effects of multiple stressors at environmentally relevant levels to elucidate mechanisms of action and interactions, and measures of interaction magnitude, for both adverse and beneficial interactions (e.g., synergism and antagonism). This would include common stressor combinations and would consider exposure influences such as sequence and timing (including for life stage) and the nature of the effects (type, level, significance, and sensitivity of response or effect severity to composition changes for the multiple stressors).
- Extrapolation methods to characterize effects from multiple stressors across various conditions (systems, species, routes, stressor types). This would include considering information from *in vitro*, animal, and other studies such as structure–activity relationships, physiologically based pharmacokinetic/pharmaco-dynamic models, neural networks, and genetic algorithms to support species, route-to-route, and duration adjustments.
- Ways to define reliable biomarkers of effect for multiple stressors. For example, this could incorporate knowledge management and statistical and visualization approaches (including bioinformatics) to link information on biomarkers of exposure from genomics through proteomics and metabolomics/metabonomics to meaningful outcomes for predicting combined effects from organisms to systems. [See Ryan et al. (2007) for additional discussion of approaches for incorporating biomarkers into cumulative risk assessment.]
- Methods to determine the biological relevance and relative significance of combined stressors and effects. For example, this would consider methods to integrate information from animal and human studies and predictive models to support effect analyses that can indicate priority combinations within and across stressor and effect types to guide analyses where those combinations exist. This would also help focus further research.
- Approaches for characterizing the severity/functional impairment and recovery/reversibility (including with treatment) of effects from various exposures to multiple stressors. This would include methods for multiple-stressor influences on homeostasis and adaptive responses.
- Methods to better characterize variability and uncertainty for cumulative assessments across multiple stressors–effects. This would include assessing differential sensitivity or susceptibility, for example, considering system components, life stage, genetic, and other factors.
- Methods to couple environmental and public health data with epidemiologic information. Tools to track, interpret, and apply these data would include considering the influence of existing diseases on susceptibility to subsequent stressors.
- Improved decision frameworks and criteria to integrate cumulative effects across types (human health, welfare, and ecologic) to guide decisions and policies for net protection. This would include qualitative and quantitative approaches that can incorporate various metrics (from biometric to econometric and medical measures) and rank combined risks for different settings or groups.

## Figures and Tables

**Table 1:** Phased effects-based approaches that account for the combined effects of multiple stressors.

Element	Rationale, methods, and tools
Step 1	
Develop conceptual model that provides insight into the stressors and the ways in which they may cause effects. In an effects-based approach there are usually a few receptors and end points that are the focus of the assessment and the bases for constructing the conceptual model.	Three levels of increasing complexity are available depending on the needs of the project and availability of resources: *a*) fixed illustrations of overall models and submodels; *b*) interactive models that contain content on the nature of the relationships; *c*) interactive electronic- hierarchical models such as those used to develop Bayesian networks.
Establish common denominators for the assessment; this involves identifying common receptors and end points for evaluation.	If the effect involves an exposure group or an area defined by geography, GIS-based approaches can be helpful for organizing and evaluating the spatial information and can support the development of the conceptual model.
Step 2	
Screen stressors to arrive at an appropriate and manageable number for the problem at hand.	This can be accomplished by comparisons with reference values and reference conditions, by using candidate lists and look-up tables for familiar problems, and through expert elicitation and discussions with stakeholders.
Step 3	
Evaluate the individual effects of individual stressors, as there may be a predominant stressor that is contributing or could contribute to an effect.	Apply stressor identification, life course, and epidemiologic concepts for effects-based approaches. Associations or lack of associations are evaluated through statistical analyses and evaluating available information by applying epidemiologic principles. Reliance is also placed on scientific literature and on laboratory and/or field studies designed to test particular hypotheses about causality. Correlation and regression analyses can be used to inform the evaluation about the potential importance of an individual stressor. GIS and other mapping approaches can be used to visualize the spatial relationships between the observed effects and the potential stressors.
Step 4	
Evaluate the combined effects of stressors without considering the potential for interactions.	The analysis in the preceding step may reveal that the effects can be only partially explained by any one stressor and that a combination of stressors is contributing to the observed effect. An example of stressors that contribute directly to an effect but that do not interact is given for striped bass. Statistical tools such as multiple and logistic regression, and process models can be used to explore the contributions of various stressors to defined receptors and end points, and to help explain and predict stressor–effect relationships. GIS and other mapping approaches can be used to visualize the overlay of stressors with the observed effects.
Step 5	
Evaluate the combined effects of stressors, taking into account potential interactions among the stressors and effects.	This level of analysis would be undertaken if previous analysis reveals that important interactions exist wherein one stressor affects another. Knowledge reflected in conceptual models would provide a starting place for describing these potential interactions. Matrix approaches would provide a means of visualizing the nature of the potential interactions. Interactions can also be visualized and evaluated using response surfaces and by building influence diagrams and Bayesian networks. Factorial multiple analyses of variance can be useful for identifying interactions.

**Table 2:** Utility of selected multivariate statistical applications for addressing multiple stressors.

Statistical method	Utility for evaluating combined effects
Factorial multiple ANOVA	Can be used to identify interactions among stressors and among effects. Therefore, it is a useful method for exploring whether combined effects are occurring.
ANCOVA	Can be used to isolate the effects of a particular stressor. Therefore when there are multiple stressors under consideration, this methodology can help determine whether specific ones are important. The method cannot be used to examine interactions among stressors.
Regression analyses	Can be used to evaluate the contributions of individual stressors to the observed effects. The resulting regression equations can be used to predict the effects of the combined stressors. Relationships could be linear or nonlinear. While the regression equation can be a useful predictor for the system from which it was derived, its reliability diminishes when applied to systems or conditions that are outside the bounds of the original system.
Canonical correlation analysis	Can be used with continuous data to examine the relationship of several measures of effects to a suite of stressors. A major limitation is the method’s inability to assess interactions among the effects or the stressors.
Multiway frequency analysis	Can be used to examine relationships among three or more discrete variables. The method relies on a chi-square type approach to predict in which group a new case belongs. The approach is flexible and can be applied to a large number of study designs.
Logistic regression analysis	Can be used to predict a discrete outcome (e.g., disease/no-disease) based on input of multiple stressors and other environmental variables. The method can accommodate variable data types and can account for nonlinear relationships. The method ascertains whether there is a relationship between any of the stressors or environmental attributes alone or in some combination and the measured effects. Like multiple regression, this approach can produce predictive models.
Discriminate function analysis	Can be used to provide information on the predictive power of various stressors or environmental attributes for explaining groupings of effects. Typically, most of the predictive power is captured by two or three variables. The approach is most suitable when data sets are of similar types. Considerable knowledge of the system (ecologic or human) is required to make effective use of this approach.
Nonmetric cluster analysis	Can be used to identify relationships when data sets are of different types. The method produces clusters of variables that tend to be intuitively obvious and amenable to interpretation. Groups are distinguished using as few variables as possible with simple “yes/no” comparisons. An iterative approach is used to associate the cluster of effects with identified stressors. Quantitative or qualitative information can be used for the stressors.
Principal components analysis	Can be used to identify groups of stressors or environmental attributes that contribute most to the observed effects. The goal is to reduce the complexity of the problem to a few components that can explain underlying processes. This is often used as an exploratory tool and requires good knowledge of the system for interpretation.
Cluster analysis	Can be used as a data exploration tool to group stressors with respect to observed effects or conditions. The method imposes a structure on the data set that can provide insight into important groupings (clusters). However, because the method will always impose some type of structure, knowledge of the system is needed to evaluate whether the formed clusters are meaningful.

From Fairbrother and Benett (2000).

**Table 3:** Iterative stressor-based approaches that account for the combined effects of multiple stressors.

Element	Rationale, methods, and tools
Step 1	
Develop conceptual model that provides insight into the stressors and the ways in which they may cause effects. In a stressor-based approach, there may be a few or many possible stressors that are under evaluation. Identify the receptors and end points that may be affected by the stressors individually or in combination. As with an effects-based approach it is most useful to establish common denominators for the assessment.	The types of approaches are similar to that for effects-based approaches. The main difference is that the development of the model begins with the stressors and considers how receptors might be affected through direct or indirect effects and combinations. In some cases the assessment may be focusing on a stressor known to combine with or interact with other stressors. In such cases, these stressors need to be represented in the conceptual model. In other cases, the assessment may be exploring whether important combinations or interactions might exist. In that case, care must be taken to identify all potential stressors. For new or poorly understood stressors, the development of Bayesian networks may be helpful for organizing information and exploring possible relationships among stressors and how they may combine to affect receptors. Matrix-based approaches, including the relative risk model (RRM), can be helpful for structuring the conceptual model and laying a foundation for analyses of relative risks associated with stressors and combinations of stressors.
Step 2	
Screen stressors of interest, determining which need to be included in the assessment and which may act in combination.	This can be accomplished by identifying groups of stressors that are known or suspected to act additively or to interact in some other fashion. Look-up tables can be helpful to check for insights or guiding principles across types of combinations and potential interactions. Matrix approaches including RRM can be used to establish some initial rankings of stressors to evaluate which ones should be carried further in the analysis. Using the RRM in this way can also guide the gathering of subsequent information. Uncertainties associated with stressors or combination with higher ranks would receive more attention and resources than stressors with lower ranks or with less uncertainty.
Step 3	
Evaluate the individual effects of individual stressors of interest along with combinations with other stressors. As part of developing the conceptual model (including discussions with experts and stakeholders), common denominators framed in terms of receptors and end points should be selected as the focus of the evaluation, mindful of the management decision to be made. The analysis of combined effects of multiple stressors will be directed on how they collectively influence the end point. Psychosocial stressors can be incorporated into the analysis by characterizing the environmental setting and the cultural and socioeconomic attributes of the exposure group.	Simple additive approaches can be used for combinations of stressors (e.g., chemical or physical) that are believed to act additively. Well-defined assessment end points are critical for evaluating combined effects of stressors for more complex problems. Tools that can be helpful include statistical models, process models, and matrix approaches including RRM. For systems that have been studied adequately to develop multiple regression or logistic models, application of these models within the bounds of the system or to similar systems can be useful for evaluating combinations of stressors that were included in the development of the statistical models. However, as conditions depart from those used to derive the models, increasing uncertainty is introduced. In such cases, process models may be more useful because they provide a mechanistic basis for evaluating how various stressors might combine to cause effects. Matrix approaches including RRM can provide a good framework for examining how stressors might together affect a particular receptor. These are relative risk metrics and do not provide a magnitude of actual (or absolute) risk. However, they can indicate where combinations of stressors are likely to be most important. GIS and other mapping approaches can be used to visualize the spatial relationships among estimates of combined risks, relative risk metrics from RRM, and the spatial distribution of exposure groups or areas and resources of interest. GIS supported by statistical and process models as is incorporated in Alternative Futures Analyses can be especially useful.
Step 4	
Evaluate the combined effects of stressors, taking into account potential interactions among the stressors and effects.	This level of analysis is an expansion upon the previous step. Knowledge reflected in conceptual models would provide a starting place for describing potential interactions. Matrix approaches provide a means of visualizing the nature of potential interactions. Influence diagrams and Bayesian networks can be used to incorporate existing knowledge on interactions. Statistical analyses of available data can be used for stressors that may be interacting.
